# MiCrowd: Vision-Based Deep Crowd Counting on MCU

**DOI:** 10.3390/s23073586

**Published:** 2023-03-29

**Authors:** Sungwook Son, Ahreum Seo, Gyeongseon Eo, Kwangyeon Gill, Taesik Gong, Hyung-Sin Kim

**Affiliations:** 1 Graduate School of Data Science, Seoul National University, Seoul 08826, Republic of Korea; 2 School of Electrical Engineering, Korea Advanced Institute of Science and Technology, Daejeon 34141, Republic of Korea

**Keywords:** crowdcounting, tiny machine learning, computer vision

## Abstract

Microcontrollers (MCUs) have been deployed on numerous IoT devices due to their compact sizes and low costs. MCUs are capable of capturing sensor data and processing them. However, due to their low computational power, applications processing sensor data with deep neural networks (DNNs) have been limited. In this paper, we propose *MiCrowd*, a floating population measurement system with a tiny DNNs running on MCUs since the data have essential value in urban planning and business. Moreover, *MiCrowd* addresses the following important challenges: (1) privacy issues, (2) communication costs, and (3) extreme resource constraints on MCUs. To tackle those challenges, we designed a lightweight crowd-counting deep neural network, named MiCrowdNet, which enables *on-MCU inferences*. In addition, our dataset is carefully chosen and completely re-labeled to train MiCrowdNet for counting people from an *mobility view*. Experiments show the effectiveness of MiCrowdNet and our relabeled dataset for accurate on-device crowd counting.

## 1. Introduction

Recently, microcontrollers (MCUs) are ubiquitously found on most electronic devices. Microcontrollers are extremely small computers that can run relatively simple computations. Because MCUs are targeted for a single application, MCUs are usually configured with lower computational processors, making them cost-efficient. Due to its cost efficiency, the MCU market is rapidly growing and is expected to grow to USD 58.2 billion by 2030 [1]. In fact, more than 29 billion units were shipped by 2021 alone [2]. Due to the compactness and ease of use, microcontrollers are being deployed from small devices (such as the Internet of Things (IoT)) to bigger products (such as vehicles). MCUs can be combined with cameras, inertial measurement units (IMU), and GPS sensors to collect various data that can lead to new insights.

**Figure 1:**
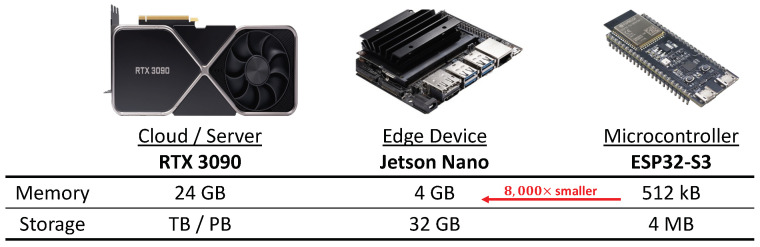
An illustration of the resource constraints of MCUs. MCUs are approximately 8000× and 48,000× more constrained in terms of memory and storage compared to Nvidia Jetson Nano and RTX 3090, respectively.

To change raw data to become meaningful, data need to be processed and analyzed. In the past, due to resource constraints on MCUs, data were sent to servers to be processed and analyzed. However, sending data to the server can create excessive communication costs and invade privacy. Recently, efforts have been made to process data on devices to tackle communication costs and privacy issues. As shown in Figure 1, MCUs suffer from extreme memory and storage limitations. Typical MCUs are equipped with memory of less than 1 MB. Due to these limitations, even a lightweight model that runs on an edge device [3], such as MobileNet [4], is impossible to run on MCUs. Thus DNN applications on MCUs have been limited to extremely simplified tasks, such as simple image and audio binary classifications.

In this work, we explore a new practical application based on tiny DNNs. Our target task is crowd counting, which counts the number of people on the image. We chose this task since it brings practical value. Specifically, we can measure the city’s floating population, and data can be used to understand citizen behaviors and facilitate urban planning and management. For example, pedestrian data that represent the number of people on each street at a given time help improve public transportation planning, store locations, and street walkability.

The traditional methodology used for floating population measurements is based on smartphone usage. Mobile network operators (MNOs) collect and analyze smartphone locations based on the cellular base stations they are connected to. The value of such data is demonstrated by the non-trivial subscription fees charged by MNOs, such as approximately USD 25,000 for hourly, single-city data for a year, as offered by KT in the Republic of Korea. However, since an MNO tracks smartphones instead of actual people [5], the accuracy of floating population measurements through MNOs is limited, in that (1) it does not differentiate between pedestrians and drivers and (2) only includes individuals subscribed to a specific MNO. In this paper, we aim to provide a fresh eye on floating population measurements by suggesting a novel floating population estimation system, utilizing MCUs embedded in electric scooters (E-scooters), a new means of micro-mobility that is spreading rapidly throughout many cities.

**Application scenario.** The E-scooter-sharing market is rapidly growing since E-scooters are cheap to rent and easy to park and drive in crowded cities [6,7]. The volume of the global E-scooter-sharing market is expected to reach USD 40–50 billion by 2025 [7]. Now that E-scooters are pervasive in many cities, if each parked E-scooter periodically measures the number of people around it and reports the information to the E-scooter vendor, the vendor can obtain floating populations in these cities. In addition, floating populations measured by E-scooters are uniquely valuable, i.e., they can focus on pedestrians, regardless of which MNO they use.

**Figure 2:**
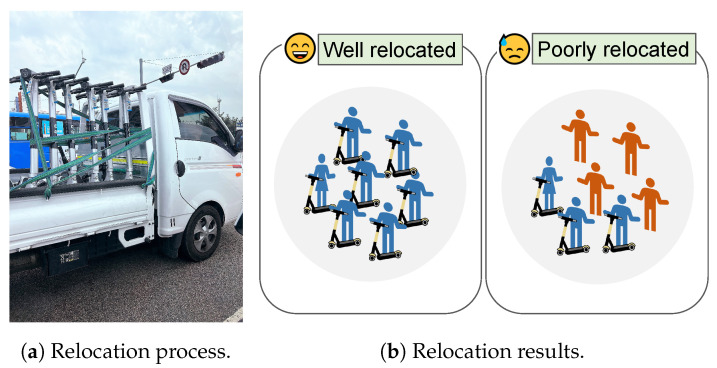
An illustration of E-scooter relocation. As collecting and relocating many E-scooters from various places requires significant costs, an E-scooter vendor should carefully determine where and how many E-scooters to relocate to improve the usage ratio to compensate the relocation costs.

On the other hand, E-scooter vendors are willing to obtain their own floating population data, not only because the data are valuable, in general, but also because these vendors have their practical target applications, i.e., *E-scooter relocation*. An E-scooter-sharing system requires high charging and management costs, which account for 62% of the revenue [7]. To maximize their profit, E-scooter-sharing companies must improve the efficiency of their E-scooter management systems. As shown in Figure 2, one representative way is to locate E-scooters at places with more potential customers so that customers can easily find an E-scooter nearby when they need it. Without its own data, however, an E-scooter company purchases the floating population data from an MNO, which is not only expensive but also not optimal for E-scooter relocation since the data include drivers who are not potential customers. Overall, when E-scooter vendors gather their own floating population datasets that focus on pedestrians, this allows these vendors to improve E-scooter relocation, remove expensive data subscription fees, and make data-related businesses based on the data.

**Figure 3:**
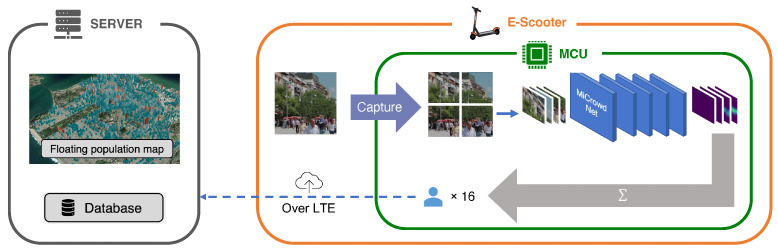
An overview of *MiCrowd*. E-scooters predict floating populations from their camera images using MiCrowdNet. The predicted population is sent to the server wirelessly and aggregated for visualization.

To this end, we devised an entire system architecture for E-scooter-based floating population measurements, called *MiCrowd*. As illustrated in Figure 3, an E-scooter takes a picture of the street nearby. Second, the picture is fed into MiCrowdNet, the deep neural network (DNN) for *MiCrowd*, to calculate the number of people appearing in the picture. Lastly, only population value and the E-scooter location are sent to the central server to minimize communication costs and privacy violations. With information from many E-scooters around the city, a city-wide floating population map can be derived. Note that our proposal can be applied to various types of mobility, such as bicycles, and public transportation.

**Challenges.** Crowd counting, despite its value and practicality, is a relatively difficult task and it needs to count people in a noisy outdoor environment. Typically, the model becomes heavier and more complex. In this manner, most of the recent works adopted deep neural networks (DNNs) owing to their capability of understanding complex scenes and remarkable performances. As mentioned above, MCUs have extremely strict memory and storage constraints, which are not ideal for running DNNs on them. Various types of alternative sensors can be combined with MCUs, such as wireless transceivers (e.g., Bluetooth and Wi-Fi) and passive infrared (PIR) sensors. However, they are not viable options for crowd counting in outdoor environments because the signals are frequently interrupted by various noises or security settings. Sending images to the server cannot be an alternative since the communication costs are too high and it can cause privacy issues.
**Approach.** To address the resource constraints, we devised a lightweight crowd-counting DNN, MiCrowdNet, and applied *patch-based training and inference* to keep image resolution with low memory usage. In addition, we deliberately selected Person Re-identification in the Wild (PRW) dataset [8] since existing datasets for crowd counting contain congested crowds or are taken from high places [9,10,11,12], which was not the case in our mobility-based scenario. Moreover, we *completely relabeled* the dataset because PRW was originally not for crowd-counting tasks. As a result, MiCrowdNet achieves 1.12 in the mean absolute error (MAE) (comparable to state-of-the-art DNNs) with a 4× smaller model size compared to the existing lightest model [12]. It runs on Espressif ESP32-S3, a high-end microcontroller unit (MCU) of 240 MHz clock, 512 kB SRAM, and 4 MB flash.
**Contributions.** We summarize key contributions as follows:

- Our *MiCrowd* system provides a novel and practical application scenario: on-device floating population measurements with low communication/hardware costs and privacy preservation.
- To the best of our knowledge, MiCrowdNet is the first attempt to run a vision-based crowd-counting DNN on resource-constrained MCUs.
- We built a testbed to evaluate on the MCU. Our results show that MiCrowdNet is exceedingly smaller but competitive with previous models in the PRW dataset [8].
- We envision that *MiCrowd* can serve as a valuable floating population data source for other business and/or research uses.

## 2. Background and Related Work

Vision-based crowd counting involves counting people in an image with machine learning. In contrast to well-known object detection tasks, where each object in an image is labeled with a bounding box and its class name, the crowd-counting task usually utilizes a *density map* as a label for an image [12,13,14]. In a density map, a person’s head is represented using a circular Gaussian kernel instead of a bounding box. The center pixel of a Gaussian kernel has the highest value and neighboring pixels have lower values as their distance from the center increases. The sum of all values in a Gaussian kernel is one, representing that there is one person in an image.

Crowd-counting models can be categorized into two groups: (1) multi-column CNN-based models, and (2) single-column CNN-based models. MCNN [12] is a representative and initiative multi-column CNN-based model. It proposes a multi-column network with three branches, each of which has various sizes of CNN kernels. Following MCNN, a variety of CNN-based networks have emerged. For example, SANet [14] uses a similar multi-column network but adopts an encoder–decoder architecture, which leads to a state-of-the-art performance. On the other hand, there are single-column networks, such as CSRNet [13] and SFANet [15]. Single-column networks achieve high performance especially in congested crowd datasets as they use deeper backbone networks, such as VGG16 [16]. CSRNet, using VGG as the backbone, adopts dilated convolution to increase the receptive field. SFANet utilizes attention-based network architecture to emphasize head regions from the images.

To the best of our knowledge, the proposal in this work, *MiCrowd*, is the first vision-based crowd-counting system that both runs on the MCU and considers E-scooter-specific characteristics.

## 3. *MiCrowd*

### 3.1. Design Principles

**Vision-based Crowd Counting.** In *MiCrowd*, an E-scooter takes a picture, which is fed into MiCrowdNet, a vision-based DNN. In fact, several sensors can be considered for crowd counting. For example, since smartphones have Bluetooth Low Energy (BLE) radios, counting the number of beacons received nearby may represent the number of pedestrians. However, smartphones broadcast BLE beacons only when requested by the user for security reasons, which makes smartphones unviable for crowd counting. A passive infrared (PIR) sensor array might be another option since it has been used for counting occupants [17,18]. However, PIR-based techniques work only in delicately designed indoor environments since the raw PIR signal is notoriously noisy [19]. For these reasons, we chose images as our data sources, which contain dense data and do not suffer from the aforementioned issues in alternative sensors.
**On-MCU Inference.** Our target scenario expects E-scooters to periodically capture photos on the street while being parked. However, sending photos that capture pedestrians without their consent not only raises privacy concerns but also results in significant communication overhead. Thus, our primary goal in devising a DNN is to make it lightweight enough to support on-MCU inference to mitigate those issues. As our target-embedded platform, we selected Espressif ESP32-S3, one of the widely-used high-end MCUs that runs at 240 MHz with 512 kB SRAM and 4 MB flash, considering previous work on E-scooters that utilized ESP32 variants [20,21].

### 3.2. Dataset Considering Mobility View

**Figure 4:**
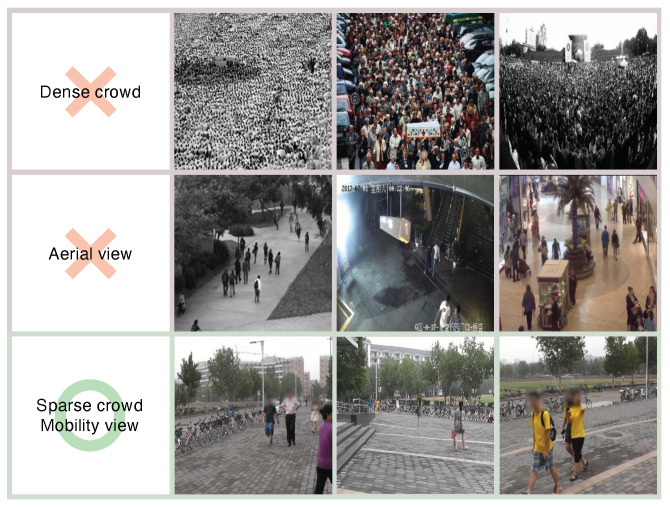
Qualitative analysis of the difference between existing datasets and our target datasets (Person ReID in the Wild).

Constructing a proper dataset for training and testing is essential to generate a DNN that works for practical applications. In *MiCrowd*, we assume that (1) pictures are taken at a *mobility view* (i.e., from 1∼1.5 m in height), and (2) pictures contain a *sparse crowd* (i.e., at most 10–20 people). Existing datasets for crowd counting [9,10,12] do not fit the application scenario because they have a significantly dense crowd (e.g., hundreds of people) or are taken from an aerial view, as illustrated in Figure 4. Instead, we chose the Person Re-identification in the Wild (PRW) dataset [8], which consists of pictures that are taken from a human-eye view (similar to the *mobility view*) and contain 3.64 people, on average, and 19 people, at maximum (i.e., sparse crowd).

**Relabeling Process.** For a crowd-counting task, it is necessary to create a ground-truth density map for each image, which requires detecting each person’s head position and applying a Gaussian kernel at its center point. However, the PRW dataset, originally built for pedestrian detection and person re-identification, contains bounding boxes of people (i.e., the entire body) but not those of their heads.

**Figure 5:**
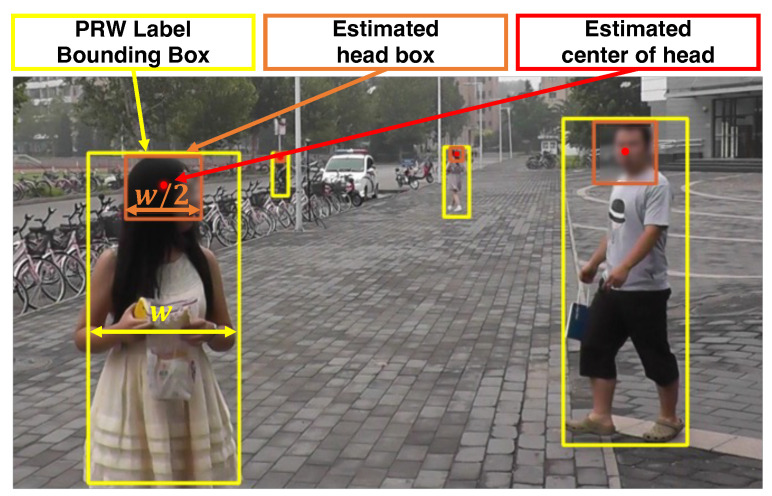
An illustration of head approximation for the relabeling process.

To avoid relabeling all 11,816 images in the PRW manually, we adopted a simple estimation of the head position. As shown in Figure 5, we auto-labeled the point at the horizontal center and 1/10th of the height from the top as the center point of the head for each bounding box containing a person. The intuition is that human heads are located approximately 1/10th of the height and in the middle of the width of the entire body, as shown in a statistical analysis [22]. After relabeling, input images were resized to 640 × 480 resolution, which many IoT cameras support. Density maps were also resized into 160 × 120 resolution according to the MiCrowdNet architecture.

**Adaptive Gaussian Kernel Sizes.** One of the crucial challenges in crowd-counting tasks is the variation in head sizes [23]; using a fixed Gaussian kernel for different head sizes would be detrimental to the performance. This is because DNN would capture the size variations of the heads through training, while a fixed Gaussian kernel does not have any information about the size of each head. To address the issue, each head’s kernel width should be proportional to the head size.

**Figure 6:**
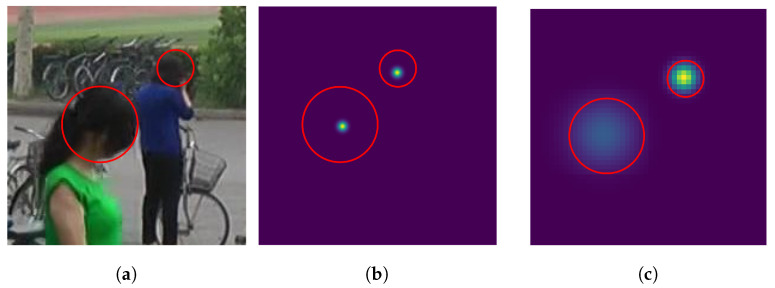
Ground-truth density maps for an image, with and without adaptive-sized kernels. The adaptive kernels capture various head sizes better. (**a**) Image. (**b**) Density map (baseline). (**c**) Density map (adaptive kernel—ours).

In contrast to previous studies that estimated head sizes using unsupervised segmentation [24], we estimated the head size utilizing bounding-box labels for entire bodies, which are given by PRW. Specifically, inspired by [22], which shows statistically that the human head width is approximately 1/2∼1/3 of the body width, we created bounding boxes for the heads accordingly. Figure 6 shows the density map with a fixed kernel size in Figure 6b and adaptive kernel size in Figure 6c from the same input image in Figure 6a. With this technique, the circles in the density map properly represent the actual head sizes in the input image.

### 3.3. MiCrowdNet

**Figure 7:**
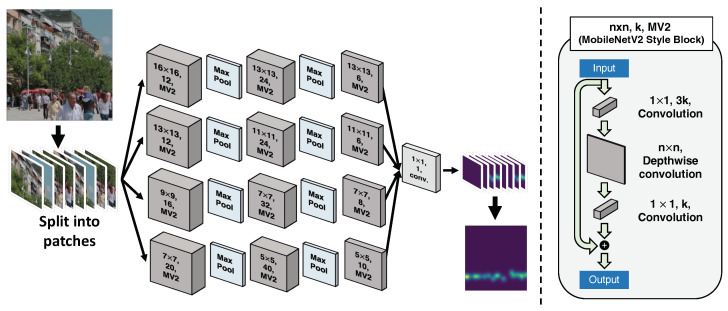
Architecture of MiCrowdNet. An input image is sliced into patches. The dark gray blocks represent the MobileNetV2 (MV2) style blocks [25]. MV2 consists of three sequential convolution layers. After passing the network, slices of the density map are produced.

We designed MiCrowdNet, which is optimized for running on the resource-constrained MCU to obtain crowd counts from the mobility view. Figure 7 describes the architecture of MiCrowdNet. Further implementation details are as follows.

**Optimizing for Mobility View.** In contrast to common crowd-counting scenarios that utilize images taken from a high place, we aim to count people in images taken from human-eye levels; conventional crowd-counting DNNs might not work in this particular scenario. Specifically, the variations of head sizes are larger as pedestrians can be close to or far from parked E-scooters. To capture the *multi-scale features* simultaneously, MiCrowdNet has *four branches of different kernel sizes.* Compared to the popular MCNN [12], which considers images taken from high places, MiCrowdNet has one more branch and bigger kernel sizes since the heads in our target dataset (i.e., mobility view) are bigger. The biggest kernel in MCNN [12] is a 9 × 9 kernel, whereas that in MiCrowdNet is 16 × 16.
**Minimizing the Model Size.** For on-MCU inference, it is essential to reduce the size of the model since the previous crowd-counting vision models are often too large to fit in the MCU’s memory (Table 1). To this end, we leverage *only three layers for each branch*, which is one layer fewer than MCNN, to capture multi-scale features with a smaller number of parameters and peak memory usage. Our intuition is that the previous crowd-counting models, including the original MCNN, are designed to detect a group of people who appear small in high-resolution images, while our scenario involves counting people who appear relatively larger and, thus, are present in smaller numbers. Therefore, we speculate that our task requires less computation compared to the other crowd-counting models, and removing a layer from MCNN may not significantly affect the model’s performance, but potentially result in faster and more efficient crowd-counting.

To further reduce inference time, we adopt *MobileNetV2 convolution block*, motivated by MobileNetV2 [25]. Lastly, both the weights and activations of MiCrowdNet are quantized into 8-bit integers after training. We used TensorFlow Lite [26] to apply post-training full quantization. These modifications contribute to the small number of parameters and accelerate on-MCU inference.

**Minimizing the Input Size (Patch-based Operation).** To enable on-MCU inference, it is necessary to reduce not only the model size but also the input image resolution. Thus, we propose dividing the image into smaller *patches* and feeding them to MiCrowdNet one by one; both training and inference are based on a patch rather than an entire image. In our case, the resolution of an original image is 640 × 480, and it is split into non-overlapping 80 patches where each patch is 64 × 64 in resolution. Although complex patch-based techniques can improve accuracy [27], we adopted a simple slicing technique that could run on the MCU.

**Table 1:** Performance of various crowd-counting DNNs on the PRW dataset. Models were trained and tested using patches of 64 × 64 from 640 × 480 images.

Model	Number of Params.	Model Size	MAE
P2PNet [28]	6.86 M	82.4 MB	**1.00**
CSRNet [13]	16.26 M	65.1 MB	1.14
MCNN [12]	0.13 M	543 kB	1.57
**MiCrowdNet (float)**	**0.07 M**	309 kB	1.08
**MiCrowdNet (quantized)**	**0.07 M**	**137 kB**	1.12

### 3.4. Testbed for On-MCU Inference

**Figure 8:**
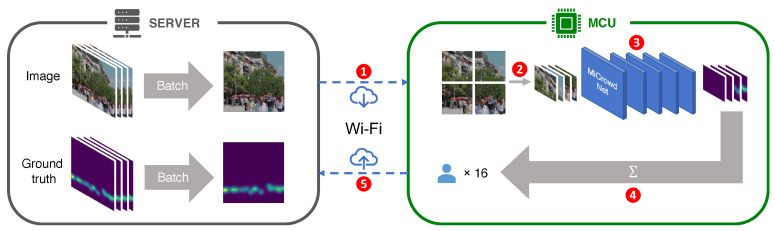
Our testbed to validate inferences on the resource-constrained MCU (ESP32-S3).

We built a testbed to measure the on-MCU inference performance of MiCrowdNet, as illustrated in Figure 8. Since an MCU cannot store the whole test dataset locally, we implemented the server that stored the test dataset and sent the images to the MCU one by one for inference. Specifically, the testbed operates for each test image as follows: (1) The server sends a test image to the MCU wirelessly (Wi-Fi). (2) The MCU divides the image into smaller patches. (3) The MCU loads each patch into memory and performs inference. (4) The MCU produces the result for the entire image by accumulating inference results for all patches. (5) The MCU sends the final result for the image to the server wirelessly. (6) The server evaluates the prediction results for the image.

## 4. Evaluation

We evaluated the MiCrowdNet performance on PRW, using 5704 images for training and 6112 images for testing, as in [8]. When training images and test images, we followed the official training and test data separation from the PRW dataset. We used the mean absolute error (MAE) as the accuracy metric, which intuitively tells how close the prediction value is to the ground truth. Formally, MAE is defined as:

$$MAE=\frac{1}{N}\sum_{i=1}^{N}\left|y_{i}-{\hat{y}}_{i}\right|,$$

where *N* is the number of test samples, $y_{i}$ is the ground truth count, and ${\hat{y}}_{i}$ is the predicted count from the model on the image. For example, if the model has an MAE of 1, the prediction count of the model is off by one on average. For the embedded platform, we use ESP32-S3, which has a 240 MHz clock, 512 kB SRAM, and 4 MB flash.

**Comparison with Baselines.** To compare MiCrowdNet with existing crowd-counting models, we selected baselines for our experiment: CSRNet [13], P2PNet [28], and MCNN [12], all trained on PRW for 64 × 64 patch-based inference. Table 1 shows the results in terms of the number of parameters, model size, and MAE.

**Figure 9:**
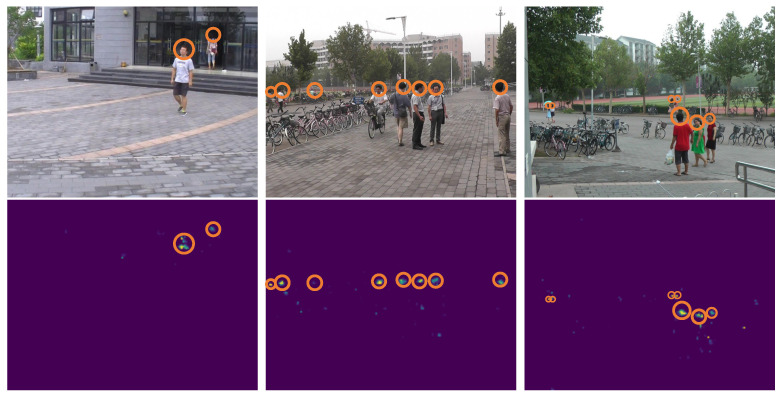
Qualitative analysis of MiCrowdNet. MiCrowdNet accurately creates density maps (second row) from the images (first row).

As shown, MiCrowdNet is significantly smaller in size compared to the baselines, 600× smaller than the heaviest P2PNet and even 4× *smaller than the lightest MCNN*. With this compact architecture, MiCrowdNet provides 1.12 MAE, is 29% more accurate than MCNN, and is comparable to other much heavier models (P2PNet and CSRNet). This verifies the effectiveness of our design choices for MiCrowdNet: one more kernel and one less layer than MCNN, MobileNetV2 blocks, and quantization. Qualitative analysis of MiCrowdNet in Figure 9 also shows that MiCrowdNet can detect people and generate density maps accurately.

**Impact of Quantization.** Given that our target application scenario involves MCUs, it is critical to consider the limited resources of these devices. As most MCUs lack floating point units, floating point operations are typically emulated, which creates a significant processing burden. To address this issue, we applied quantization to the model, which reduces the precision of the model’s weights and activations.

To evaluate the effectiveness of the quantization approach [26,29] and understand the latency in different platforms, we conducted experiments using three platforms: Intel Xeon Gold 5120 (CPU), Nvidia Tesla V100 (GPU), and ESP32-S3 (MCU). The results, presented in Table 2, indicate that the MiCrowdNet (quantized) exhibited a significant improvement in performance on the MCU compared to the MiCrowdNet (float) version. Specifically, the MiCrowdNet (float) model took approximately 12.6 s to execute on the MCU, while the MiCrowdNet (quantized) model was completed in approximately 5.7 s, representing a 2.2× speedup. This highlights the effectiveness of quantization in optimizing the performance of resource-constrained devices, such as MCUs.

**Impact of Patch Sizes.** Table 3 shows the impact of patch sizes on the MiCrowdNet performance. Using the whole 640 × 480 image requires ∼10 MB in memory, far exceeding the memory constraint of ESP32-S3. It verifies that patch-based inference is essential to run MiCrowdNet on MCU. In addition, Table 3 shows that the larger the input size, the slower the inference for each patch and the larger the peak memory. On the other hand, input sizes that are too small could lead to severe performance degradation. Considering the trade-off, we chose a patch size of 64×64 for MiCrowdNet as in Section 3.3.

**Figure 10:**
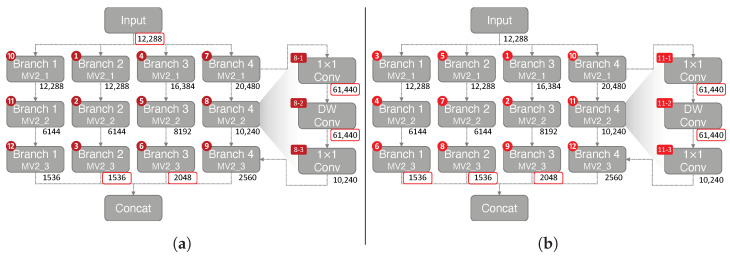
Demonstration of the difference between the order of operation. The red numbers labeled on the top left of each MV2 block indicate the order of operations. The numbers below the blocks indicate the tensors stored in memory in bytes during the peak memory operation. The sum of the tensors on memory demonstrates the peak memory. For a patch size 64 × 64, reordering the operations reduces peak memory by **11.4%**. For illustration purposes, some orders have been simplified. (**a**) TensorFlow Lite default ordering (Peak memory: 135.5 kB). (**b**) Memory-optimized ordering (Peak memory: 125 kB).

**Impact of Reordering Operations.** When performing convolutional operations, the MCU needs to allocate memory space for the input and output tensor to perform calculations. Furthermore, when a tensor is reused for future operation, the tensor must be stored in memory to perform calculations. The tensor can be removed only after no future operation exists using the tensor. Figure 10a shows the order of operations when using MiCrowdNet on TensorFlow Lite. The peak memory occurs when performing the DW Conv on Branch 4 MV2_2 block (operation 8-2 in Figure 10a. This is due to the greater output channel (40) compared to other blocks. The tensors stored during peak memory for TFLite default ordering (Figure 10a) are the input tensor, Branch 2 MV2_3, Branch 3 MV2_3, 1 × 1 Conv (operation 8-1), and DW Conv (operation 8-2). The sum of these tensors equals 135.5 kB.

To reduce peak memory, we implemented a reordering operation algorithm based on [30], which uses a dynamic programming algorithm to traverse the graph of operations to optimize peak memory usage. The optimized ordering is shown in Figure 10b, which further saves peak memory by 11.4%. On the optimized ordering, the peak memory also occurs when performing the DW Conv on Branch 4 MV2_2 block (operation 11-2 in Figure 10b). The tensors stored during the peak memory for memory-optimized ordering are Branch 1 MV2_3, Branch 2 MV2_3, Branch 3 MV2_3, 1 × 1 Conv (operation 8-1), and DW Conv (operation 8-2).

The difference between the two orderings is the execution order of Branch 4. In the memory-optimized ordering, Branch 4 is executed last, after all other branches. However, in the default ordering, Branch 1 needs to be executed after Branch 4. Therefore, for the TensorFlow Lite default order, the input tensor needs to be in memory to execute Branch 1 (operations 10–12), which places a greater burden on memory. In the memory-optimized ordering, Branch 1 is calculated before Branch 4, allowing the input tensor to be removed from memory.

**Impact of Camera Viewpoints.** Next, we evaluate the importance of a dataset that reflects the E-scooter view. We used ShanghaiTech [12], a popular dataset for crowd counting (high-place view), and CSRNet [13], a heavy DNN for crowd counting. For ShanghaiTech, we used 400 images for training and 316 images for testing, as in [12]. Since we focus only on accuracy in this experiment, the models were trained and tested on full-resolution images.

In Table 4, both MiCrowdNet and CSRNet show significant performance degradation when trained on one dataset and tested on the other dataset. Although CSRNet is 480× heavier and can contain much more knowledge, its knowledge (learned from an aerial view) becomes useless when the data domain is shifted at the test time. On the other hand, when trained on our relabeled PRW, MAE is *more than* 100× *reduced*. This verifies that *camera viewpoint does matter*: a different viewpoint causes a significant data domain shift, which impacts the test accuracy regardless of the model architecture.

## 5. Discussion and Future Work

**Optimal Camera Placement.** As our focus is to measure the floating population with a single camera attached to a scooter, proper camera placement is important to cover a population of interest. Note that the effectiveness of camera placement largely depends on the lens used. In this study, the OV2640 camera was considered, which has a coverage range between 68${}^{\circ }$ to 160${}^{\circ }$.

Using multiple cameras could be an ideal solution for expanding the coverage at the cost of purchasing and maintaining multiple cameras as well as inferencing multiple images. Finding an ideal number of cameras and their placement in terms of the angle between them and their height requires further investigation. To optimize camera placement, it is essential to strike a balance between comprehensive monitoring and cost-effectiveness. The results of this study can be used as a basis for future research on optimal camera placement for effective monitoring via scooters.

**More Realistic Dataset.** We assume that the PRW dataset is similar to real-world data in terms of the number of people, angles, and sizes of the heads. Nevertheless, real-world data might have differences from the PRW dataset. For example, the PRW dataset covers limited weather and time conditions. In future work, we will collect our own real-world dataset to reflect more diverse outdoor conditions using E-scooters with low-cost IoT cameras and MCUs.
**Multi-scooter Collaboration.** It has become increasingly common for E-scooters to be parked in designated areas to avoid disturbing pedestrians. If the people counts from E-scooters in the same location are similar, running MiCrowdNet on all of these E-scooters may be redundant. Investigating this hypothesis could be an interesting area for future research. If it is true, E-scooters can be clustered using GPS information and only a representative of the cluster can run MiCrowdNet to reduce communication costs and save the battery‘s lifetime. The cluster leader might also offload some patches to the cluster members to run MiCrowdNet in parallel. Otherwise, if neighboring E-scooters report different people counts, several cluster members that have different camera angles can be selected to improve accuracy.

## 6. Conclusions

Floating population data are invaluable but hard to obtain because of their extensive scales. This work is the first and novel attempt to gather floating populations using E-scooters scattered over a city. We propose *MiCrowd* to tackle privacy, communication costs, and resource constraints on MCUs. Specifically, we provide a dataset that is proper for E-scooter viewing and an on-MCU crowd-counting DNN called MiCrowdNet. Experiments show that MiCrowdNet improves MAE with a 4× smaller model size compared to MCNN, the lightest DNN for crowd counting. We hope this work will provide a fresh outlook on micro-mobility, as a new sensor for a smart city. Finally, it is important to note that although this paper mainly describes an E-scooter-based scenario, the essence of this work is on-MCU vision-based crowd counting that is not bounded by the E-scooter application.

## Figures and Tables

**Table 2:** MiCrowdNet inference latency comparison between CPU, GPU, and MCU. For comparison, we used the TensorFlow 32-bit float model and the quantized TensorFlow Lite 8-bit model. The input size was set as a 64×64 image.

	CPU Xeon Gold 5120	GPU Nvidia V100	MCU ESP32-S3
MiCrowdNet (float)	0.26 s	0.035 s	12.6 s
MiCrowdNet (quantized)	0.13 s	-	5.7 s

**Table 3:** Memory requirement and inference time of MiCrowdNet according to different patch sizes. OOM represents out-of-memory (MAE is still reported by calculating at the server).

Patch Size (Pixels)	Number of Patchs	Peak Mem. (kB)	Inference Time (s/patch)	MAE
32 × 32	300	31.25	1.3	1.28
64 × 64	80	125	5.7	1.12
128 × 128	20	500	(OOM)	1.04
640 × 480 (Whole image)	1	9375	(OOM)	1.03

**Table 4:** Evaluation results (MAE) with various test and training datasets that show the impact of the domain shift.

Model	Train Dataset	Test on PRW	Test on Shanghai B
CSRNet	Shanghai B	99.86	10.67
CSRNet	PRW	0.96	101.49
CSRNet	Shanghai B + PRW	0.95	65.05
MiCrowdNet	Shanghai B	113.24	18.92
MiCrowdNet	PRW	1.03	103.33
MiCrowdNet	Shanghai B + PRW	1.02	84.98

## Data Availability

Publicly available datasets were analyzed in this study. This data can be found here: http://zheng-lab.cecs.anu.edu.au/Project/project_prw.html accessed on 10 January 2023.
